# Setback distances for unconventional oil and gas development: Delphi study results

**DOI:** 10.1371/journal.pone.0202462

**Published:** 2018-08-16

**Authors:** Celia Lewis, Lydia H. Greiner, David R. Brown

**Affiliations:** 1 Southwest Pennsylvania Environmental Health Project, New Haven, Connecticut, United States of America; 2 Southwest Pennsylvania Environmental Health Project, McMurray, Pennsylvania, United States of America; University of Pittsburgh, UNITED STATES

## Abstract

Emerging evidence indicates that proximity to unconventional oil and gas development (UOGD) is associated with health outcomes. There is intense debate about “How close is too close?” for maintaining public health and safety. The goal of this Delphi study was to elicit expert consensus on appropriate setback distances for UOGD from human activity. Three rounds were used to identify and seek consensus on recommended setback distances. The 18 panelists were health care providers, public health practitioners, environmental advocates, and researchers/scientists. Consensus was defined as agreement of ≥70% of panelists. Content analysis of responses to Round 1 questions revealed four categories: recommend setback distances; do not recommend setback distances; recommend additional setback distances for vulnerable populations; do not recommend additional setback distances for vulnerable populations. In Round 2, panelists indicated their level of agreement with the statements in each category using a five-point Likert scale. Based on emerging consensus, statements within each category were collapsed into seven statements for Round 3: recommend set back distances of <¼ mile; ¼—½ mile; 1–1 ¼ mile; and ≥ 2 mile; not feasible to recommend setback distances; recommend additional setbacks for vulnerable groups; not feasible to recommend additional setbacks for vulnerable groups. The panel reached consensus that setbacks of < ¼ mile should not be recommended and additional setbacks for vulnerable populations should be recommended. The panel did not reach consensus on recommendations for setbacks between ¼ and 2 miles. The results suggest that if setbacks are used the distances should be greater than ¼ of a mile from human activity, and that additional setbacks should be used for settings where vulnerable groups are found, including schools, daycare centers, and hospitals. The lack of consensus on setback distances between 1/4 and 2 miles reflects the limited health and exposure studies and need to better define exposures and track health.

## Introduction

In the oil and gas extraction industry hydraulic fracturing, the injection of a mixture of water, chemicals, and sand under high pressure, has increased rapidly since the late 1990s. Critics have voiced concerns about long-term potential impacts on air, water, and soil quality that may accompany hydraulic fracturing and all stages of the processes associated with the development and transport of produced oil and gas (i.e. unconventional oil and gas development or UOGD) [1–9]. Additional concerns include the significant impact on surrounding communities caused by increased traffic, light, noise, and social disruption from this type of industrial development [10–13]. The entire process of UOGD, including oil and gas discovery, drilling, production, processing, waste management, and transport, includes many sources of air and water pollution, presenting risk factors for the environment, human health and community social structure.

### Health and proximity to UOGD activity

Several recent studies have documented health outcomes related to closer proximity to UOGD activity. Steinzor, et al. [14], in their descriptive community study, documented increasing numbers of symptoms reported by residents as proximity to any type of UOGD facility decreased. Rabinowitz et al. [15] conducted a cross-sectional study to investigate the relationship between proximity to unconventional gas wells and reported health symptoms in a random sample of 429 residents of 180 households that had ground-fed water wells. GPS readings were taken at each household as residents completed a health survey. ArcGIS was used to calculate the distance of the home from natural gas wells. In this study, the number of symptoms reported per individual increased with household proximity to wells. Within 1 kilometer (km) of wells, residents reported more skin and respiratory symptoms compared to residents who lived at a greater distance.

Mckenzie et al. [16] estimated health risks for two populations in the Garfield County, Colorado gas fields: residents living less than or equal to 1/2 mile away from gas wells and those greater than ½ mile. They found that the populations living closer to gas wells were at higher risk of respiratory, neurological, and other health impacts and had a higher lifetime risk for cancer than those who lived at farther distances. For this study ambient air samples were collected from a fixed monitoring station located near unconventional natural gas development and residences, and from locations at the perimeters of four well pads. Methodology used by the Environmental Protection Agency were used to estimate non-cancer Hazard Indexes and excess lifetime cancer risks for exposures to hydrocarbons.

In a retrospective cohort study of 124,842 births in Colorado between 1996 and 2009, Mckenzie and colleagues [17] found an association between congenital heart defects and proximity and density of unconventional natural gas wells within 10 miles of maternal residence, using inverse distance weighted natural gas well counts as a measure of proximity and density. Results also suggested a possible association between neural tube defects and proximity and density. In another retrospective cohort study, Casey et al. [18] examined the relationship between exposure to unconventional gas development and birth outcomes in 10,946 births in Pennsylvania between 2009 and 2013. Unconventional gas development was modeled using distance from residence; dates of well pad preparation, drilling and hydraulic fracturing; and amount of production during pregnancy. Results showed an association between increased exposure and preterm birth, but no association between low APGAR scores, small for gestational age, or low term birthweight. Stacy and colleagues [19] also used an inverse distance weighted gas well count to examine the relationship of exposure to birth outcomes in their retrospective cohort study of 15,451 births in southwestern Pennsylvania between 2007 and 2010. Results showed increased exposure was associated with low birth weight and small for gestational age; it was not associated with preterm birth.

Tustin et al. [20] used self-reported symptoms to investigate associations between chronic rhinosinusitis, migraine, and fatigue, three conditions frequently reported in communities exposed to UOGD. Responses to self-report questionnaires were reviewed using standard criteria. Exposure was estimated using an “activity index” [18] derived from four exposure metrics to account for different phases of well construction and production: distance from the residence; timing of well pad development, drilling, and hydraulic fracturing; and volume of gas produced. Results of the case-control analysis indicated that the highest quartile of the activity index was associated with increased odds of all three outcomes, when compared with the lowest quartile.

McKenzie et al. [21] investigated the relationship between acute lymphocytic leukemia and non-Hodgkin’s lymphoma in children ages 0–24 and residential proximity to unconventional oil and gas development in Colorado. Cases and controls (i.e., children diagnosed with non-hematologic cancers) were diagnosed between 2000 and 2013 during rapid expansion of UNGD. Exposure was calculated using an inverse distance weighted (IDW) approach, first described by McKenzie et al. [17], to count all active oil and gas wells within 16.1 miles of each residence, giving greater weight to those that are closer. In the adjusted model, acute lymphocytic leukemia cases age 5–24 were 4.3 times likely to live in the highest well-count tercile as controls, with a monotonic increase across IDW tertiles (p for trend = 0.035). No such relationship was seen in leukemia cases 0–4 years or in non-Hodgkin’s lymphoma cases of any age.

Rasmusen and colleagues [22] conducted a nested case-control study to investigate the relationship between asthma exacerbations and exposure to unconventional natural gas development. Using the Geisinger Clinic electronic health records, they identified cases of mild (i.e., new medication prescribed), moderate (i.e., emergency department visit), and severe (i.e., hospitalization) asthma exacerbations (n = 20,749; 1,870; and 4,782 respectively) treated at Geisinger between 2005 and 2012. Exposure was measured using the activity metric previously described by Casey [18]. In the adjusted model, mild, moderate, and severe asthma exacerbations were associated with high scores in each activity metric when compared to referents.

### Setback distances and UOGD

A 2013 review of state setback distances for shale gas development shows the broad range of regulations in place at the time [23]. Of the 31 states in the review, 20 had setback restrictions specifically from buildings, 11 had none related to buildings. The restricted distances ranged from 100 feet (NY) to 1,000 feet (MD). California required setbacks, not from buildings but between wells and public roads. For this type of land-based restriction, the American Petroleum Institute recommended that “…the wellsite and access road should be located as far as practical from occupied structures and places of assembly” [24], offering a simple discretionary guideline. Setback restrictions for water sources were found in 12 states; 18 had none and one state had a discretionary standard. The regulated distance from water sources varied from 50 feet (OH) up to 2,000 feet (NY). A review of setback distances in urban areas of the Texas Barnett Shale showed a similarly broad range of regulations [25]. While the State permitted drilling within 200 feet of a dwelling, most municipalities employed longer distances; in Denton County these ranged from 300 to 1500 feet. Fry also found that 12 out of the 26 city setback ordinances reviewed had increased the distance over time–and none had been decreased. The author found that setback restrictions appeared to be politically rather than technically-based decisions and recommended greater reliance on “advanced emissions monitoring” to minimize discrepancies in determining appropriate setback distances.

Several authors have examined potential exposures related to existing setback distances. McCawley [26] conducted a study of air, noise and light impacts using the West Virginia state setback distance of 625 feet from the center of well pads. Measurable levels of dust and volatile organic chemicals, including one or more of benzene, toluene, ethylbenzene, and xylene, were found at all seven drilling sites where measurements were taken. Some benzene concentrations were above the “minimum risk level” for no health effects. Dispersal patterns were influenced by factors including multiple sources of emissions located throughout the well pad, local weather, topography, and wide fluctuation in levels of contaminants. Light levels, measured as skyglow, were zero during night time; ionizing radiation levels measured from filtered airborne particulate were near zero as well. While average noise levels calculated for the duration of work at each site were not above the 70 dBA level recommended by the EPA, the noise at some locations was above that allowed by EPA regulation for vehicles engaged in interstate commerce and local noise ordinances. McCawley concluded that a setback distance of 625 feet cannot assure that nearby residents would not be exposed to drill site contaminants.

Haley et al. [27] reviewed current regulations and other aspects of setback distances used within the Marcellus, Barnett, and Niobrara shale plays. The most common setback distances from buildings were 300 and 500 feet, with a range of 150 to 1500 feet. The authors concluded that current setback distances are inadequate to protect residents in the case of explosions, radiant heat, toxic gas clouds, and air pollution from hydraulic fracturing activities; and that setback distances cannot provide absolute measures of safety, especially for vulnerable populations.

There is an increasing number of peer-reviewed articles addressing air quality impacts from UOGD (see for instance Physicians, Scientists and Engineers for Healthy Energy database) [28]. While these studies provide valuable science-based data that can support the rationale for regulating or not regulating setback distances, there remains a concern about the adequacy of health-based standards used to determine impacts from pollutant exposures.

In a critique of current methods of collecting air emissions data, Brown et al. [29] found that data collection and analysis of air pollution impacts from unconventional natural gas development cannot accurately assess human health impacts near UOGD sites. Specific findings were that “1) current protocols used for assessing compliance with ambient air standards do not adequately determine the intensity, frequency or durations of the actual human exposures to the mixtures of toxic materials released at UOGD sites; 2) the typically used periodic 24 hour average measures can underestimate actual exposures by an order of magnitude; 3) reference standards are set in a form that inaccurately determines health risk because they do not fully consider the potential synergistic combinations of toxic air emissions; 4) air dispersion modeling shows that local weather conditions are strong determinates of individual exposures.” The authors recommend protocols that provide continuous chemical monitoring to show variations in exposure; modeling of local weather conditions to identify periods of high exposures; and sampling for chemical mixtures to identify the major components.

Two examples of air modeling studies provide context for assessing the need for setback distances. Olaguer [30] used a neighborhood scale dispersion model to simulate ozone formation resulting from emissions from UOGD in the Barnett Shale, focusing on both routine and nonroutine emission events (flares). The model predicted that both types of UOGD operations can have a significant impact on local ambient ozone levels. Modeled ozone levels increased at an approximate distance of 2km or more, at enhancement levels greater than 3 parts per billion (ppb). Modeled flare events could cause greater increases at distances >8km downwind. Ozone causes respiratory health effects including asthma and chronic obstructive pulmonary disease (COPD).

In another study, Brown et al. [31] describe a hypothetical case that demonstrates the direct effect of weather on exposure patterns of particulate matter (specifically PM_2.5_) and volatile organic chemicals (VOCs) from unconventional natural gas infrastructure. The authors modeled the frequency and intensity of exposures to PM_2.5_ and VOCs at a residence surrounded by three UOGD facilities. The hypothetical well pad, compressor and processing plant are 1 km, 2 km and 5 km distant from the residence. Modeled peak PM_2.5_ and VOC exposures (defined as 2 standard deviations above the mean) during 14 months of well development occurred 83 times. Modeled compressor station emissions created 118 peak exposure levels and a gas processing plant produced 99 peak exposures over one year. The authors emphasize that local weather patterns combined with episodic emissions drive local exposure profiles.

While there is emerging evidence that proximity to UOGD activities is associated with chemical exposures and health outcomes, there is intense debate about “How close is too close?” The Delphi is an accepted method for reaching convergence of expert opinion about a specific topic, particularly when available data are inconclusive [32]. We conducted this Delphi study to arrive at expert consensus on two closely related questions: 1) the relationship between health outcomes and UOGD activities; and 2) appropriate setback distances for UOGD from human activity including residences, schools, work places, and farms. This paper reports the expert consensus on the question of appropriate setback distances; expert consensus on the question of relationship between health outcomes and UOGD activities will be presented in a subsequent report. Portions of this report on setback distances have been issued as a technical report by Southwest Pennsylvania Environmental Health Project www.environmentalhealthproject.org

## Methods

### Study design

This study used a conventional Delphi procedure [32–35], which can be viewed as a series of rounds. In each round, the participants (called “panelists”) respond anonymously to a set of questions and then receive information about the responses of all other participants, including their own. Panelists are encouraged to re-assess their own responses on subsequent rounds with a goal of reaching consensus. The first round consists of a set of open-ended questions. Subsequent rounds consist of a set of statements to which panelists indicate their level of agreement on a five-point Likert scale. Three rounds are usually sufficient to reach consensus [35]. For this study consensus was defined as agreement of 70% of panelists, a decision point that is frequently used in Delphi studies [36–38].

### Expert panel

There are few generally accepted criteria for inclusion on a Delphi panel [34] or agreement about the number of panelists required for a Delphi [39]. Early researchers who used this technique suggested the following criteria for inclusion: background and experience with the topic, capability to contribute, and willingness to revise their judgment to reach consensus [40]. More recent researchers suggest identifying stakeholders with interest in the topic: positional leaders, authors of publications in the scientific literature, and those with first-hand experience [41,42]. As Keeney et al. point out in their critical review of the technique, the definition of “expert” ranges from informed individuals to experts in the field [43]. The number of panelists required varies with the focus of the Delphi and the characteristics of the panelists. Generally, the more similar the members and the more narrow the focus of the investigation, the smaller the number, with 10–15 generally considered acceptable if the group is homogeneous; 15–30 if it is heterogeneous [43].

For this Delphi panel, selection criteria included: researchers whose work has been published in peer-reviewed journals and/or presented at national scientific meetings; scientists employed in regulatory agencies; and leaders in public policy and environmental advocacy who have been published in the grey literature. Potential panelists included representatives of federal and state agencies, environmental advocacy groups, health care providers, public health practitioners, and a range of researchers in areas including environmental science, toxicology, and social science. Invitations were sent via e-mail or the US Postal Service if no e-mail address was publicly available. The invitation included a consent to participate and the first round questions, along with an estimate of time commitment for participation. The study was reviewed and approved by the Duquesne University Institutional Review Board.

A total of 57 experts were invited to participate in this Delphi; 18 agreed to be panelists and returned the completed Round 1 questionnaire and consent form. Of those who did not participate, 23 simply did not respond to the invitation. A total of 18 provided a reason for declining, citing lack of time (n = 7), lack of expertise (n = 8), and no longer working in UOGD (n = 2).

### Round 1

In the first round, panelists were asked to respond to the open-ended questions shown in Table 1, following these instructions:

“We are interested in both gas and oil and know that the multiple steps in the production of these products differ. We understand that a panelist may have more expertise in one area than the other, so have constructed questions to allow for those differences. Where possible in your responses, please address all steps in the process from drilling site construction through delivery of the product to the consumer (e.g., well pad construction, well drilling, hydraulic fracturing, compressor stations, pumping stations, processing plants, impoundments, pipelines, and other steps in the process). In the questions below, the steps in this process are referred to as ‘related activities’.”

**Table 1:** Open-ended questions used in Round 1.

**1**	*What do you believe are appropriate set-back distances for hydraulic fracturing and related activities from places where people live*, *including single homes*, *multiple family dwellings*, *etc*.? *Please specify if your response is related to oil or gas extraction*.
**2**	*What do you believe are appropriate set-back distances for hydraulic fracturing and related activities from indoor places where people work including offices*, *hospitals*, *and schools*? *Please specify if your response is related to oil or gas extraction*.
**3**	*What do you believe are appropriate set-back distances for hydraulic fracturing and related activities from outdoor places where people work such as farms*? *Please specify if your response is related to oil or gas extraction*.
**4**	*What do you believe are appropriate set-back distances for hydraulic fracturing and related activities from places where people recreate or play such as parks*? *Please specify if your response is related to oil or gas extraction*.
**5**	*Should set-back distances differ for settings that include groups of vulnerable individuals*, *such as schools*, *day care centers*, *long- term care facilities*, *and if so*, *how*? *Please specify if your response is related to oil or gas extraction*.

Five open-ended questions were sent to all prospective panelists for their responses to initiate Round 1 of the Delphi study.

Panelists were asked to return their responses within two weeks. Non-responders were sent a reminder at the end of two weeks. For those who requested additional time due to workload, travel, etc. the deadline was extended two weeks. The same procedure was followed in subsequent rounds.

### Round 1 data analysis and development of Round 2 structured questionnaire

Content analysis was conducted on the qualitative responses to the open-ended questions in Round 1, with all responses independently coded by two members of the research team (CL and LG). Coding was compared for congruence. Similar responses were grouped into categories, for example, “Recommended setback distances” and “Cannot recommend setback distances” as shown in the Results section. Within the category “Recommended setback distances” responses were grouped into mutually exclusive sub-categories. Responses to the question concerning vulnerable populations were grouped into two categories; both are shown in the Results section. All responses in each category were included on the structured questionnaire used for Round 2 and 3.

The structured questionnaire for Round 2 included all responses so that each panelist was able to see the complete range of responses in each category, with his/her own responses highlighted. Panelists were asked to indicate their level of agreement with each statement using a 5-point scale: strongly agree, agree, not sure, disagree, and strongly disagree and to provide a rationale for their decisions for those statements for which they strongly agreed or agreed.

### Round 2 data analysis and development of Round 3 structured questionnaire

Responses to Round 2 were used to revise the structured questionnaire for Round 3. Statements within categories were collapsed to reflect emerging consensus within the panel. The Round 3 questionnaire provided the aggregated panelists’ responses for each statement and the rationales provided by the individual panelists for their responses. For this final round, panelists were asked to review the distribution of responses and rationales provided and then indicate their level of agreement with each statement.

## Results

### Characteristics of panelists

The 18 panelists who agreed to participate and completed Round 1 self-identified as researchers/scientists, health care providers, environmental advocates, and public health practitioners. Self-reported areas of expertise included: medicine/health care, air quality, water quality, toxicology, environmental science, environmental health, public health, epidemiology, social science, policy, and risk analysis. The majority (83%) of the panelists hold earned doctoral degrees and reported working in their respective fields for a mean of 17.6 years (SD = 10), with a range of 4–35 years. In the area of UOGD specifically, they reported a mean 4.3 years (SD = 1.2), with a range of 2–6 years. The panelists represented a range of geographic regions throughout the United States; 50% were women. None of the authors participated as panelists. Of the 18 panelists, 14 (78%) participated in Round 2 and18 (100%) participated in Round 3.

### Round 1

Responses to Questions #1- #4 were similar, with 9 panelists providing word-for-word the same response to all four open-ended questions. An additional four panelists provided the same response to three of the four questions. Only two panelists provided a different response to each of the four questions of setback distances from home, places of work, and places of recreation. Thus, all responses to these questions were considered together in the content analysis; two categories of responses, shown in Table 2, emerged.

**Table 2:** A comparison of exemplar statements recommending setback distances and exemplar statements not recommending setback distances from homes, places of work, or recreation areas.

**Recommended setback distances**
*I defer to existing regulation*: *Center of well pads may not be located within 1/10 mile (0*.*1 km) of an occupied dwelling structure*.
*2/10 mile (0*.*3 km) for gas operations based on industry studies of blowouts*, *explosions and fires from drill rigs*, *compressor stations and pipelines*.
*Set-backs of at least 1/3 mile (0*.*5km) would be needed to prevent flow through documented pathways of subsurface contamination*.
*½ mile (0*.*8 km) for oil or natural gas extraction from office buildings and other indoor areas*.
*Minimum of 1 mile (1*.*6 km) for gas extraction*
*1 ¼ mile (2 km) from natural gas wells*
*At least 2 miles (3*.*2 km)*, *maybe more*
**Cannot recommend setback distances**
*Due to our inability*, *with current information*, *to predict dispersal pathways accurately*, *I do not think safe set-back distances can be determined*.
*This is something that is difficult to determine because it depends on the hydrology and air currents*.
*My response applies to both oil and gas*. . . .*do not take a position on specific distances*, *in large part because there is no scientifically definitive distance beyond which health impacts would never occur*. *However*, *we believe that current setbacks from residential areas are much too short in all states*.
*I do not have an opinion on an appropriate set-back distance because I don’t believe there is enough evidence to inform an opinion*.
*Again the distinction between oil and gas is not important*. *I think there are appropriate*, *science based setbacks that could be developed*. *I agree with the position that the ones that exist are not science based at all…and are based on political compromises*.
*There are no appropriate set-back distances for recreation areas near oil production*. *Ambient air quality is affected by VOCs*. *We have no proof of what constitutes a safe set-back distance*. *Cumulative effects have yet to be studied*.

There were 17 statements that included recommendations for specific setback distances from homes; places of work such as schools, office buildings, and farms; and recreational areas. Table 2 shows recommended distances ranged from 1/10 of a mile (0.1 km) to 2 miles (3.2 km). There were 18 statements that did not include recommendations for specific setback distances. The exemplar statements in the Table 2 section “Cannot recommend setback distances” reflect panelist’s perspectives that there is insufficient information available to make recommendations. As one panelist pointed out, his lack of a specific recommendation did not imply that setback distances were not needed, just that he did not think it was possible to make a recommendation. All statements in each category were included on the structured questionnaire used for Round 2.

The content analysis revealed that responses to the question concerning setback distances for vulnerable populations differed from those to the first four questions. As shown in Table 3, panelist’s responses fit into one of two categories: responses that argued for additional setback distances and responses that focused on the difficulties of establishing setback distances for vulnerable populations.

**Table 3:** A comparison of exemplar statements recommending additional setback distances for vulnerable populations and exemplar statements not recommending additional setback distances for vulnerable populations.

**Panelists recommend additional considerations for vulnerable populations**
*Populations that are particularly sensitive to the toxins known and suspected to be associated with fracking activities should have special protections; this includes children*, *neonates*, *fetuses*, *embryos*, *pregnant women*, *elderly individuals*, *and those with pre-existing medical or psychological conditions*.
*I would consider this a case where additional restrictions would be important*. *Oil and/or gas operations near hospitals and schools should simply not be allowed…*
*Yes*, *greater setback distances are warranted for schools*, *daycare centers*, *long-term care facilities*, *etc*. *for both oil and gas extraction*.
*Larger setback distances in gas extraction are critical to larger vulnerable groups because one must take into consideration evacuation time and route in case of a catastrophic well or related infrastructure event*.
*Setbacks (gas) should definitely be farther from schools*, *day care centers where children are located and long-term facilities where people who already have compromised health don't need it further compromised by poor air quality from unconventional gas development*.
**Panelists do not recommend additional considerations for vulnerable populations**
*I am really unsure as to how to answer this because if air plumes travel and contribute to quality degradation of an entire region*, *it is likely that it would impact vulnerable populations regardless of physical proximity*.
*Regarding different set-backs for settings with vulnerable populations*: *Probably not*. *It appears that the most vulnerable populations are pregnant women and those with asthma*, *neither of which would necessarily be concentrated in specific facilities*.
*Vulnerable populations are distributed throughout the environment*. *This is therefore an inadequate calculation to consider*.
*The distances mentioned above are set to protect vulnerable persons as they are all a significant part of every society*.
*It makes sense to start with…longer setbacks on places used or inhabited by people with known vulnerabilities*. *However*, *there may be vulnerable individuals living*, *working*, *and spending time outdoors even in locations that are not specifically geared toward that population (for example*, *individuals with compromised immune systems*, *a history of cancer*, *or asthma)*.

Eleven statements recommended additional setback distances for vulnerable populations. Vulnerable populations were defined by panelists to include: children, neonates, fetuses, embryos, pregnant women, elderly individuals, those with pre-existing medical or psychological conditions, and those with pre-existing respiratory conditions. Panelists included the following settings as places where vulnerable populations might be concentrated: schools, day care centers, hospitals, and long-term care facilities. Five statements focused on the difficulties of setting additional setback distances. As shown on Table 3, the panelists focused on the distribution of vulnerable individuals throughout the population, making the determination of setback distances to protect all vulnerable members of society difficult if not impossible.

The four categories of responses described above, and all statements within each, were used to create a structured questionnaire for Round 2. Panelists were asked to indicate their level of agreement on a 5-point Likert-type scale to a total of 51 statements and to provide a rationale when they agreed with a statement. Their own statements from the first round were highlighted.

### Round 2

Based on panelist’s responses to the structured questionnaire, statements within categories were collapsed to reflect emerging consensus.

Recommended setback distances: In this category, the 17 statements were collapsed into four: less than ¼ mile; ¼—½ mile; 1–1¼ miles; and 2 or more miles. (See Table 2 for exemplar statements.) All statements fit into one of these four groups, and emerging consensus in panelists’ responses determined the cut-points used. These four statements were included on the structured questionnaire for Round 3.

Cannot recommend setback distances: Fourteen of the 18 statements were collapsed into one category which was restated as “It may not be feasible to recommend set back distances for the general population” to more accurately reflect the content of the 14 statements. (See Table 2 for exemplar statements.) For these 14 statements, the proportion of panelists who agreed ranged from 54% to 92%. Four statements were excluded because they did not reflect emerging consensus.

Panelists recommend additional considerations for vulnerable populations: Ten of the 11 statements were collapsed into one category which was restated as “Recommend additional consideration for vulnerable groups” to more accurately reflect the content of the 10 statements. (See Table 3 for exemplar statements.) The proportion of panelists who agreed with the 10 statements ranged from 58% to 83%, indicating emerging consensus. One statement was excluded because it did not reflect emerging consensus.

Panelists do not recommend additional considerations for vulnerable populations: Three of the five statements were collapsed into one category which was restated as “It may not be feasible to recommend additional considerations (i.e., members of vulnerable populations are distributed throughout the population)” to more accurately reflect the content of the three statements. (See Table 3 for exemplar statements.) The proportion of panelists who agreed with the three statements ranged from 25% - 41%. Two statements were excluded because they did not differ from the panelist’s responses to questions #1-#4.

The structured questionnaire for Round 3 included seven statements which are shown on Table 3. The questionnaire also included the distribution of panelist’s responses and their rationales offered in Round 2. Panelists were asked to review the statements and rationales and then indicate their level of agreement/disagreement with each statement on the Round 3 questionnaire.

### Round 3

The distribution of panelists’ responses to the structured questionnaire in Round 3, along with the mean and standard deviation for each statement is shown in Table 4.

**Table 4:** Distribution of panelists’ levels of agreement with statements used in Round 3 and median scores.

	1	2	3	4	5	Mean (SD)
Recommend less than ¼ mile setback	0%	0%	11%	11%	78%	4.67 (0.65)
Recommend ¼—½ mile setback	0%	11%	22%	22%	44%	4.0 (1.03)
Recommend 1–1¼ miles setback	6%	44%	28%	11%	11%	2.78 (1.05)
Recommend at least 2 miles setback	17%	17%	44%	11%	11%	2.83 (1.14)
It may not be feasible to recommend setback distances for the general population	28%	39%	6%	22%	6%	2.17 (1.09)
Recommend additional consideration for vulnerable groups	67%	22%	11%	0%	0%	1.44 (0.67)
It may not be feasible to recommend additional considerations for vulnerable groups	6%	33%	6%	33%	22%	3.17 (1.26)

1 = strongly agree; 2 = agree; 3 = not sure; 4 = disagree; 5 = strongly disagree.

To determine consensus, we combined responses of “agree” and “strongly agree” to determine the % of panelist agreement with a statement and responses of “disagree” and “strongly disagree” to determine the % panelist disagreement with a statement. Within the category “recommended setback distances”, panelists reached consensus on the statement “less than ¼ mile”. A total of 89% of panelists disagreed with that statement (i.e., 11% disagreed plus 78% strongly disagreed for a total of 89%), reaching the 70% set for consensus in this Delphi.

Panelists did not reach consensus on the statement “¼—½ mile”. For this statement, 66% of panelists disagreed with the statement, 22% were unsure, and only 11% of panelists agreed. Panelists did not reach consensus on the statement “1–1¼ miles”, 50% agreed, 28% were unsure, and 22% disagreed. Panelists did not reach consensus on the statement “at least 2 miles”; 34% agreed, 44% were unsure, and 22% disagreed. For the statement “It may not be feasible to recommend setback distances for the general population”, 67% of panelists agreed, 6% were unsure, 28% disagreed.

Regarding setback distances for vulnerable populations, panelists reached consensus on the statement “Recommend additional consideration for vulnerable groups” with 87% agreeing. Panelists did not reach consensus on the statement “It may not be feasible to recommend additional considerations for vulnerable groups”, with panelists nearly equally divided between agreement and disagreement with the statement. See S1 Chart for a visual representation of Delphi results.

## Discussion

There is significant public and scholarly debate about the relationship between proximity to these industrial activities and human health. The Delphi provides a unique tool to learn how experts on a particular topic apply their knowledge and experience to a complex problem, and to determine whether a convergence of opinion can be established [32–35, 41–43]. In this study we used the Delphi method to address the issue of appropriate setback distances for UOGD from places where humans live, work, and play. The intent of this Delphi was to reach expert consensus on appropriate setback distances from homes, workplaces, and recreation areas in general, and for vulnerable populations in particular.

The responses to the open-ended questions in Round 1 generated a set of statements that expanded the question of setback distances. The panelist’s responses reflected their opinions about the adequacy of both the evidence available to answer the question and the ability of setback distances to protect the health of the public, rather than providing simple statements of specific distances. Accordingly, their responses were grouped into four categories: recommendations for specific setback distances from places of human activity; no recommendations for specific setback distances from places of human activity; recommendations for additional setback distances for vulnerable populations; no recommendations for additional setback distances for vulnerable populations.

Round 2 responses were collapsed into seven statements, based on panelists’ responses to the individual statements and emerging consensus. Four statements focused on specific setback distances from places where people live, work, or play: *Recommend <¼ mile; Recommend ¼—½ mile; Recommend 1–1¼ mile; Recommend 2 miles or more*. Three additional statements focused on feasibility and vulnerable populations: *It may not be feasible to recommend setback distances; Recommend additional considerations for vulnerable populations; It may not be feasible to recommend additional considerations for vulnerable groups*.

### Setbacks of <¼ mile are not sufficient

Panelists reached consensus that setback distances of <¼ mile were not sufficient but were not able to reach consensus for the longer setback distances suggested by panelists (i.e., ¼—½ mile, 1–1¼ mile, and 2 miles or more). A total of 67% of panelist agreed with the statement that it may not be feasible to establish setback distances, very nearly reaching consensus. Taken together, these results suggest that while these panelists agreed that ¼ of a mile is “too close” they did not feel able to recommend a specific distance that would protect the health of the public. Failure to reach consensus about setback distances between ¼ and 2 miles reflects published studies that have identified a variety of health effects and evidence of exposure at various points within that range [14, 15, 17–22]. Nevertheless, panelists were clear that current setback regulations of less than ¼ mile are not adequate.

### Recommend additional setbacks for vulnerable populations or settings

Panelists reached consensus that additional setback distances should be established for vulnerable populations or settings. Vulnerable groups were defined by the panelists as children, neonates, fetuses, embryos, pregnant women, elderly individuals, those with pre-existing medical or psychological conditions, and those with pre-existing respiratory conditions. Vulnerable settings were defined as schools, day care centers, hospitals, and long-term care facilities. At the same time, panelists were split as to whether such consideration was actually feasible, recognizing that since vulnerable people are distributed throughout the general population it would be difficult if not impossible to give them extra consideration. Yet some suggested that where vulnerable individuals gather, such as in schools and playing fields, setbacks may be useful.

### Limitations and further research

The results of this Delphi should be interpreted with caution, as they reflect the expert opinion of one panel. It is possible that another panel would reach a different consensus, and further research is warranted. In addition, using 70% as the decision-point for consensus means that some portion of the panel is not in agreement. Therefore, we included in the results section the percentage of agreement and the mean and standard deviation of the Likert score for each statement in an effort to be as transparent as possible. While the panel had a broad range of relevant expertise in public and environmental health and many years of experience in a variety of professional activities, the panel would have been strengthened by representation from the petroleum industry. Future research should purposefully include such scientists, researchers, and practitioners. Not all panelists participated in all rounds, however, all panelists who participated in Round 1 participated in Round 3.

## Conclusion

In conclusion, the results of this Delphi study suggest that if setbacks are used the distances should be greater than ¼ of a mile from any area where human activity takes place, and that additional setbacks should be used for settings where vulnerable groups are found, including schools, daycare centers, and hospitals. The panel did not reach a consensus on setback distances between ¼ and 2 miles. While both health effects and exposures have been reported in the literature and are consistent with scientific reports, there is uncertainty with respect to levels and types of exposures and the health responses further from the wells. One report has suggested that site-specific air measures are needed. Levels of exposure have been documented based on analysis and air modeling in both air and water within ¼ of a mile. Although air modeling indicates air exposures in the ¼ to 2-mile range, it is difficult to measure due to localized weather variability. Health effects are reported in the peer-reviewed literature for respiratory disease and dermatologic effects, however the health effects could be related to the presence of other sources of pollution. Thus, failure to achieve consensus on the range of setback distances appears to reflect uncertainties based on limited data on real-time emissions from UOGD, the limited scientific studies available and the presence of periods of potential high exposures.

## Supporting information

S1 Chart Flow chart of results of Rounds 1–3 for statements that recommend setbacks for UOGD infrastructure. Consensus = 70%.
(PDF)

S1 Dataset Round 1 responses. (PDF)

S2 Dataset Round 2 responses. (XLSX)

S3 Dataset Round 3 responses. (XLSX)
