# Supplementary material for: Setback distances for unconventional oil and gas development: Delphi study results
Source: PLoS One. 2018 Aug 16;13(8):e0202462. doi: 10.1371/journal.pone.0202462 (PMC6095590; doi:10.1371/journal.pone.0202462)

Chart 1. Results of Rounds 1-3 for statements that recommend setbacks for UOGD infrastructure. Consensus = 70%

Examples of Responses for Round 1	Round 2 Results	Round 3 Results

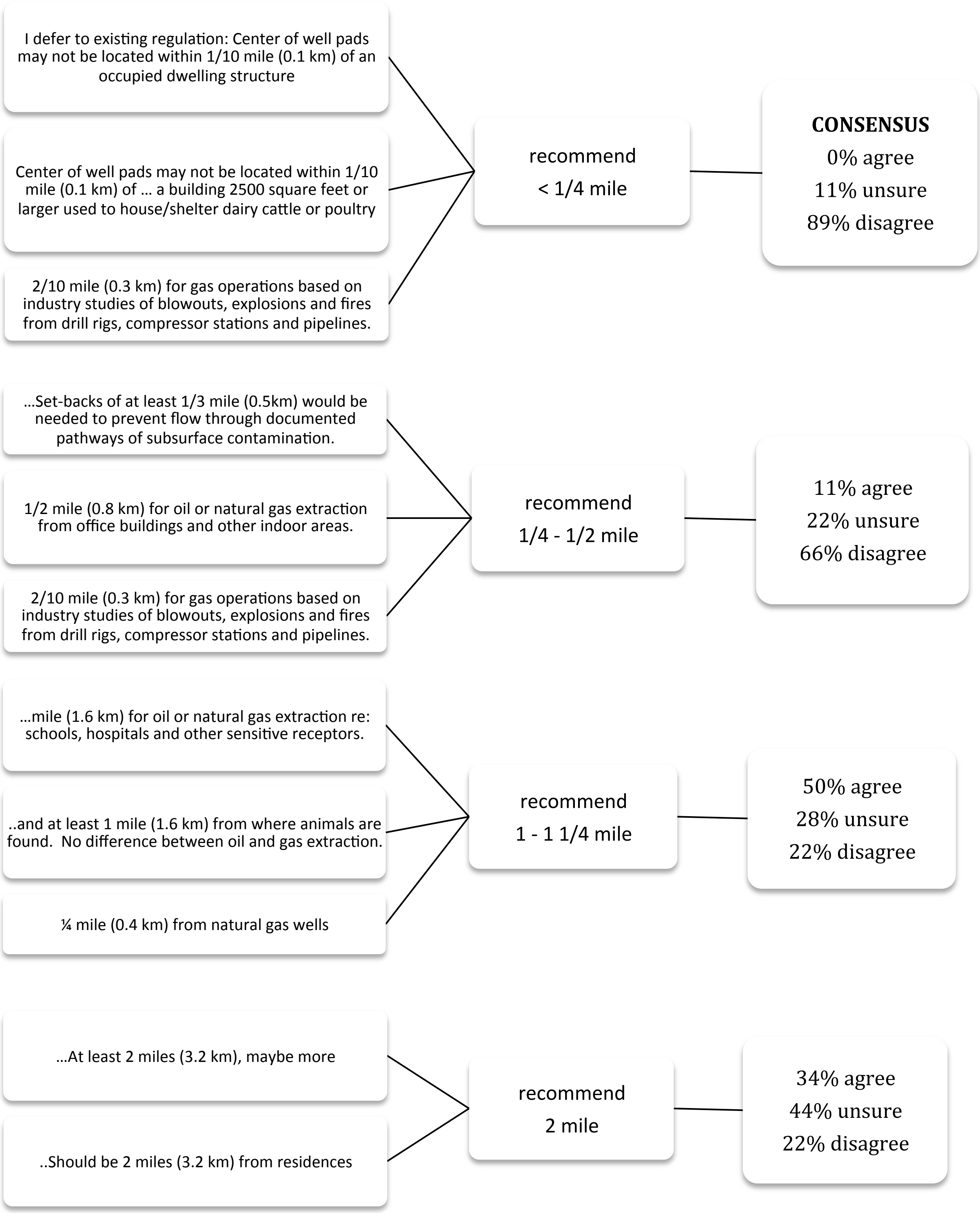

CHART 2. Results of Rounds 1-3 for statements on the feasibility of setbacks at any distance from UOGD infrastructure. Consensus = 70%

Examples of Round 1 Results	Round 2 Results	Round 3 Results

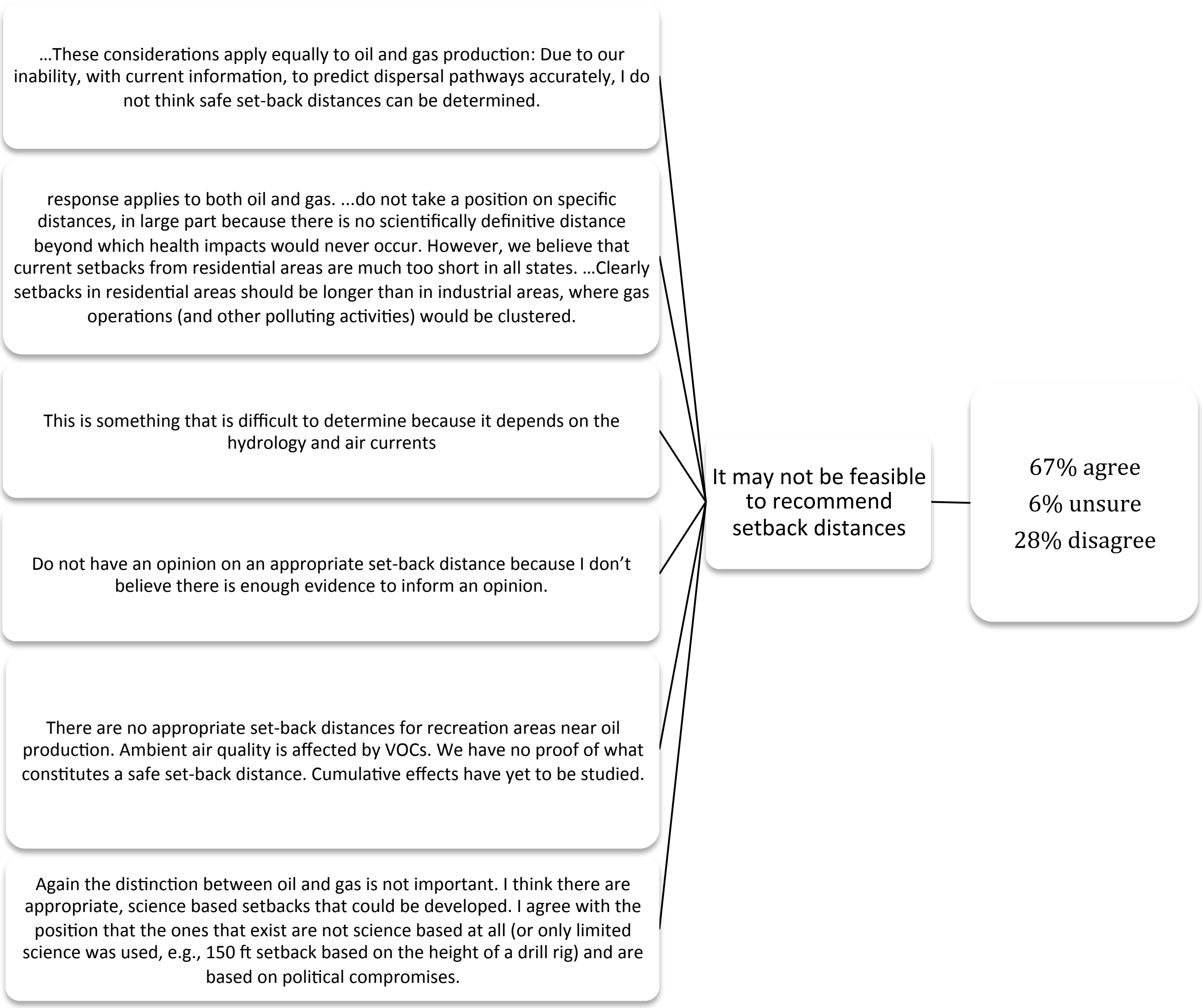

CHART 3. Results of Rounds 1-3 for statements that address the needs of vulnerable populations. Consensus = 70%

Round 1 Results	Round 2 Results	Round 3 Results

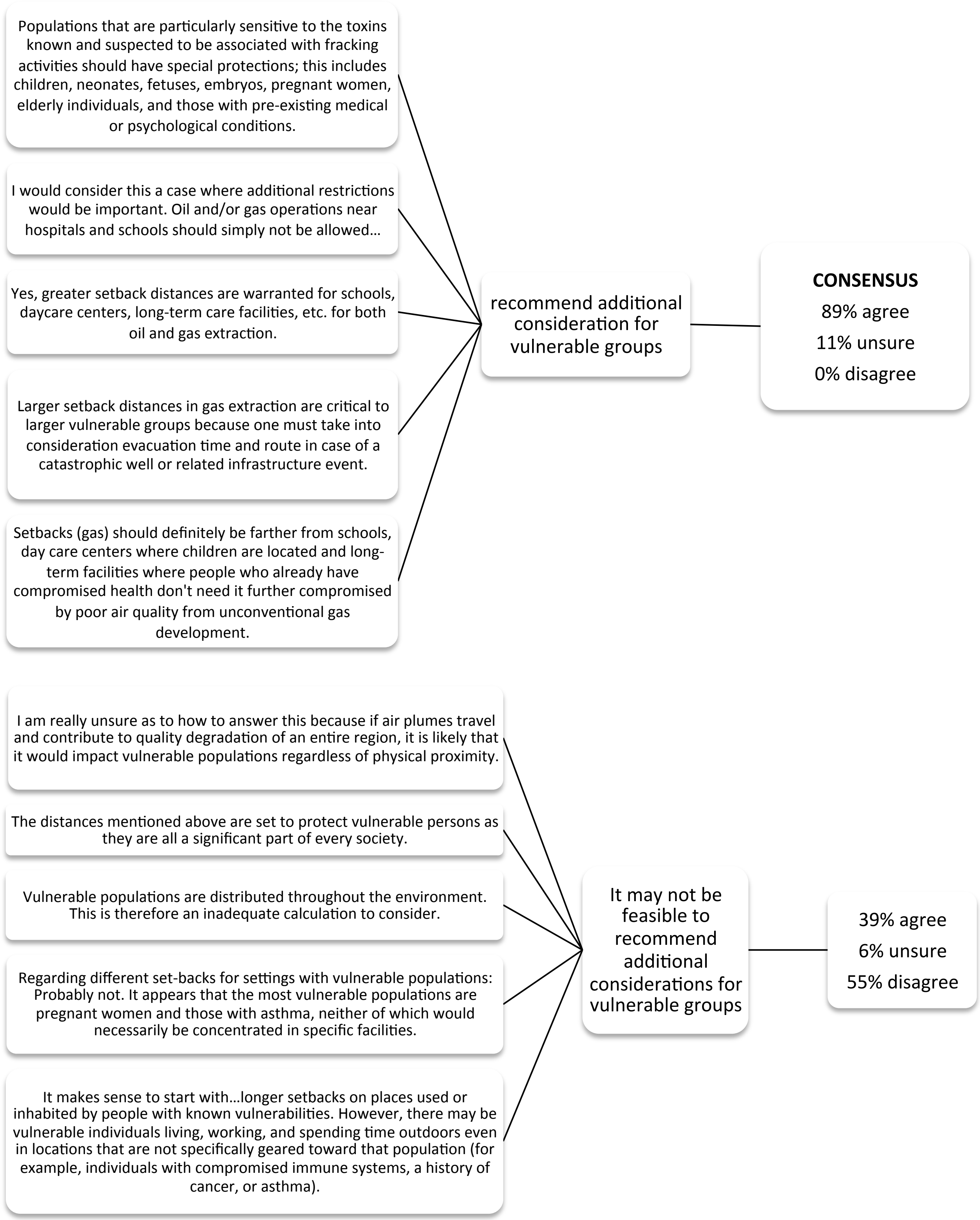

Supplement: S1 Chart — Consensus = 70%. (PDF) [file pone.0202462.s001.pdf]
