# Supplementary material for: Setback distances for unconventional oil and gas development: Delphi study results
Source: PLoS One. 2018 Aug 16;13(8):e0202462. doi: 10.1371/journal.pone.0202462 (PMC6095590; doi:10.1371/journal.pone.0202462)
Supplement: S1 Dataset — (PDF) [file pone.0202462.s002.pdf]

DA1_SetBacks

Instructions and Questions for Round One

We are interested in both gas and oil and know that the multiple steps in the production of these products differ. We understand that a panelist may have more expertise in one area than the other, so have constructed questions to allow for those differences.

Where possible in your responses, please address all steps in the process from drilling site construction through delivery of the product to the consumer (e.g., well pad construction, well drilling, hydraulic fracturing, compressor stations, pumping stations, processing plants, impoundments, pipelines, and other steps in the process). In the questions below, the steps in this process are referred to as "related activities".

As you answer the next questions keep in mind that vulnerable individuals may be found in virtually all of these settings. Vulnerable individuals may include children, adults of childbearing age, pregnant women, those with pre-existing conditions such as asthma and other chronic lung diseases, etc.

1. What do you believe are appropriate set-back distances for hydraulic fracturing and related activities from places where people live, including single homes, multiple family dwellings, etc.? Please specify if your response is related to oil or gas extraction.

   ½ mile for oil or natural gas extraction

2. What do you believe are appropriate set-back distances for hydraulic fracturing and related activities from indoor places where people work including offices, hospitals, and schools? Please specify if your response is related to oil or gas extraction.

1 mile for oil or natural gas extraction re: schools, hospitals and other sensitive receptors. ½ from office buildings and other indoor areas.

3. What do you believe are appropriate set-back distances for hydraulic fracturing and related activities from outdoor places where people work such as farms? Please specify if your response is related to oil or gas extraction.

   ½ mile for oil or natural gas extraction

4. What do you believe are appropriate set-back distances for hydraulic fracturing and related activities from places where people recreate or play such as parks? Please specify if your response is related to oil or gas extraction.

   ½ mile for oil or natural gas extraction

DA1_SetBacks

5. Should set-back distances differ for settings that include groups of vulnerable individuals, such as schools, day care centers, long- term care facilities, and if so, how? Please specify if your response is related to oil or gas extraction.

   Yes, greater setback distances are warranted for schools, daycare centers, long-term care facilities, etc. for both oil and gas extraction (see response to question 2).

Please provide the following information:

Area of professional activity (Check primary activity):

- ☐ Researcher/scientist
- ☐ Environmental advocate
- ☐ Health care provider Public
- ☐ health professional
- ☐ Other (please specify): ______________________

Area of expertise (Check primary area):

- ☐ Toxicology Air
- ☐ quality Water
- ☐ quality
- ☐ Environmentalscience
- ☐ Environmental health
- ☐ Medicine/health Public
- ☐ health Policy/law
- ☐ Epidemiology
- ☐ Social sciences
- ☐ Other (please specify): ______________________
- ☐

Number of years working in this field: 5

Number of years working on issues related to UOGD: 5

DA2_SetBacks

1. What do you believe are appropriate set-back distances for hydraulic fracturing and related activities from places where people live, including single homes, multiple family dwellings, etc.? Please specify if your response is related to oil or gas extraction.

   Hard to say given that air pollution can travel as far as 200 miles and the shale fractures can result in unpredictable pathways for water pollution. I would say there is no appropriate setback and all infrastructure carries risk. It would seem to me that compressor stations would definitely not have a safe setback because of air pollution.

2. What do you believe are appropriate set-back distances for hydraulic fracturing and related activities from indoor places where people work including offices, hospitals, and schools? Please specify if your response is related to oil or gas extraction.

I don't think my response would be different than for number 1.

3. What do you believe are appropriate set-back distances for hydraulic fracturing and related activities from outdoor places where people work such as farms? Please specify if your response is related to oil or gas extraction.

   Again, see answer #1 above. Are pipelines included in the related activities? If so, that would make it much harder to answer. To my knowledge there is no data on safe set backs from pipelines.

4. What do you believe are appropriate set-back distances for hydraulic fracturing and related activities from places where people recreate or play such as parks? Please specify if your response is related to oil or gas extraction.

   Seeing all the infrastructure is really distressing. So I would say that the setback would have to be far as not be visible from a place of recreation because that would defeat the purpose.

5. Should set-back distances differ for settings that include groups of vulnerable individuals, such as schools, day care centers, long- term care facilities, and if so, how? Please specify if your response is related to oil or gas extraction.

I am really unsure as to how to answer this because if air plumes travel and contribute to quality degradation of an entire region , it is likely that it would impact vulnerable populations regardless of physical proximity.

Duquesne University IRB
Approved: 9-22-2015
Protocol #2015-08-[illegible]
Expiration Date: 9-21-2016

DA2_SetBacks

Please provide the following information:

Area of professional activity (Check primary activity):

- ☐ Researcher/scientist
- ☐ Environmental advocate X
- ☐ Health care provider Public
- ☐ health professional
- ☐ Other (please specify): ______________________________

Area of expertise (Check primary area):

- ☐ Toxicology Air
- ☐ quality Water
- ☐ quality
- ☐ Environmental science X
- ☐ Environmental health
- ☐ Medicine/health Public
- ☐ health Policy/law
- ☐ Epidemiology
- ☐ Social sciences
- ☐ Other (please specify): ______________________________
- ☐

Number of years working in this field: 4________________

Number of years working on issues related to UOGD: 5______________

____

Duquesne University IRB
Approved: 9-22-2015
Protocol #: 2015-08-15
Expiration Date: 9-21-2016

DA3_SetBacks

1. What do you believe are appropriate set-back distances for hydraulic fracturing and related activities from places where people live, including single homes, multiple family dwellings, etc.? Please specify if your response is related to oil or gas extraction.

   At least two miles, maybe more

2. What do you believe are appropriate set-back distances for hydraulic fracturing and related activities from indoor places where people work including offices, hospitals, and schools? Please specify if your response is related to oil or gas extraction.

   Same as above

3. What do you believe are appropriate set-back distances for hydraulic fracturing and related activities from outdoor places where people work such as farms? Please specify if your response is related to oil or gas extraction.

   Should be two miles from residences, and at least one mile from where animals are found. No difference between oil and gas extraction.

4. What do you believe are appropriate set-back distances for hydraulic fracturing and related activities from places where people recreate or play such as parks? Please specify if your response is related to oil or gas extraction.

Same as above.

5. Should set-back distances differ for settings that include groups of vulnerable individuals, such as schools, day care centers, long- term care facilities, and if so, how? Please specify if your response is related to oil or gas extraction.

   The distances mentioned above are set to protect vulnerable persons as they are all a significant part of every society.

DA3_SetBacks

Please provide the following information:

Area of professional activity (Check primary activity):

- ☐ XXResearcher/scientist
- ☐ Environmental advocate
- ☐ Health care provider Public
- ☐ health professional
- ☐ Other (please specify): ____________________

Area of expertise (Check primary area):

- ☐ Toxicology Air
- ☐ quality Water
- ☐ quality
- ☐ Environmental science
- ☐ Environmental health
- ☐ XXMedicine/health
- ☐ Public health Policy/law
- ☐ Epidemiology
- ☐ Social sciences
- ☐ Other (please specify): ____________________
- ☐

Number of years working in this field: 35

Number of years working on issues related to UOGD: 5

DA4_SetBacks

Answers below are for gas extraction:

1. What do you believe are appropriate set-back distances for hydraulic fracturing and related activities from places where people live, including single homes, multiple family dwellings, etc.? Please specify if your response is related to oil or gas extraction.

This is something that is difficult to determine because it depends on the hydrology and air currents. Certainly, the original New York State proposal of 150 feet was absurd and could only have been written by someone that had never been near a gas well or someone that wanted no regulation whatsoever. Setbacks are as large as 1500 feet in other states. I would consider this to be a minimum, with additional restrictions if warranted.

2. What do you believe are appropriate set-back distances for hydraulic fracturing and related activities from indoor places where people work including offices, hospitals, and schools? Please specify if your response is related to oil or gas extraction.

I would consider this a case where additional restrictions would be important. Oil and/or gas operations near hospitals and schools should simply not be allowed, and one mile or more as a setback would not be unreasonable.

3. What do you believe are appropriate set-back distances for hydraulic fracturing and related activities from outdoor places where people work such as farms? Please specify if
your response is related to oil or gas extraction.

Farms have hosted oil and gas well throughout North America and the argument has been made that this has been the savior of the small family farm. On the other hand, farms have been lost due to contamination of farmland. We also know very little about the effects of spills and changes in air and water quality on farm animals and crops. A good case can be made for the notion that these industrial processes do not belong near food production.

4. What do you believe are appropriate set-back distances for hydraulic fracturing and related activities from places where people recreate or play such as parks? Please specify if your response is related to oil or gas extraction.

See answer #1.

5. Should set-back distances differ for settings that include groups of vulnerable individuals, such as schools, day care centers, long- term care facilities, and if so, how? Please specify if your response is related to oil or gas extraction.

See answer #2.

DA4_SetBacks

Please provide the following information:

Area of professional activity (Check primary activity):

- ☐ Researcher/scientist
- ☐ Environmental
- ☐ advocate Health care
- ☐ provider Public health
- ☐ professional

Other (please specify): ___________________________________

Area of expertise (Check primary area):

- ☐ Toxicology______ Air quality Water quality
- ☐ Environmental science
- ☐ Environmental health
- ☐ Medicine/health Public
- ☐ health Policy/law
- ☐ Epidemiology
- ☐ Social sciences
- ☐ Other (please specify):____
- ☐
- ☐
- ☐

Number of years working in this field: 30__________________

Number of years working on issues related to UOGD: 5

____

Duquesne University IRB
Approved: 9-22-2015 Protocol#2015-08-15
Expiration Date: 9-21-2016

DA5_SetBacks

- **What health outcomes do you believe are attributable to hydraulic fracturing and related activities associated with natural gas production? Please include both direct and indirect health outcomes that you believe are attributable to hydraulic fracturing and related activities. A direct health outcome might be a disease or disorder. An indirect health outcome might be a change in the environment that leads to a disease or disorder.**

- Health complications attributable to hydraulic fracturing and related activities derived from airborne chemicals, contaminated water sources or direct contact may include Respiratory, Dermatological, Constitutional, EENT, Cardiovascular, GI, Endocrine, Neurological, Musculoskeletal, Blood System and Psychological.

Direct health outcomes, which are not easily measured, may include stress, worry and anxiety. Indirect changes in the environment such as constant noise, lights, vibrations and common nuisances play a large role in the health and wellbeing of the residents as well.

- **What health outcomes do you believe are attributable to hydraulic fracturing and related activities associated with oil production? Please include both direct and indirect health outcomes that you believe are attributable to hydraulic fracturing and related activities. A direct health outcome might be a disease or disorder. An indirect health outcome might be a change in the environment that leads to a disease or disorder.**

- Although I am not as familiar with the oil production process as I am with the natural gas, I presume that the structure and development is generally similar and therefore the potential health outcomes will be similar as well.

As you answer the next questions keep in mind that vulnerable individuals may be found in virtually all of these settings. Vulnerable individuals may include children, adults of childbearing age, pregnant women, those with pre-existing conditions such as asthma and other chronic lung diseases, etc.

- **What do you believe are appropriate set-back distances for hydraulic fracturing and related activities from places where people live, including single homes, multiple family dwellings, etc.? Please specify if your response is related to oil or gas extraction.**

- Placing set back distances on natural gas activities may not be able to be as general as one would hope. Simply saying oil and gas development should be 1 mile from any population might not be entirely true. Setback distances may need to be done by looking at each source on an individual basis as each process (drilling, transporting, compressing, processing, etc.) would offer its own concentration and potential distance of pollution.

Each source will also have its own 'identity' such as what part of the process is it, what are the potential pollution sources (air, water or both), how close to a population it is, what is the surrounding topography and many other characteristics.

- DA5_SetBacks
- **What do you believe are appropriate set-back distances for hydraulic fracturing and related activities from indoor places where people work including offices, hospitals, and schools? Please specify if your response is related to oil or gas extraction.**

- **What do you believe are appropriate set-back distances for hydraulic fracturing and related activities from outdoor places where people work such as farms? Please specify if your response is related to oil or gas extraction.**

- **What do you believe are appropriate set-back distances for hydraulic fracturing and related activities from places where people recreate or play such as parks? Please specify if your response is related to oil or gas extraction.**

- **Should set-back distances differ for settings that include groups of vulnerable individuals, such as schools, day care centers, long- term care facilities, and if so, how? Pleasespecify if your response is related to oil or gas extraction.**

- Sites that include vulnerable individuals should be taken into consideration. Adding an additional setback distance to schools, day care centers and long-term care facilities should be a priority among the considerations of all setback distances. At this time, there is not enough information available to make an educated estimate as to what that additional distance should be.

DA5_SetBacks

Please provide the following information:

Area of professional activity (Check primary activity):

- ☐ Researcher/scientist
- ☐ Environmental advocate
- ☐ Health care provider
- ☐ Public health professional
- ☐ Other (please specify): ____________________

Area of expertise (Check primary area):

- ☐ Toxicology
- ☐ Air quality
- ☐ Water quality
- ☐ Environmental science
- ☐ Environmental health
- ☐ Medicine/health
- ☐ Public health
- ☐ Policy/law
- ☐ Epidemiology
- ☐ Social sciences
- ☐ Other (please specify): ____________________

Number of years working in this field: 5 Years

Number of years working on issues related to UOGD: 3 ____________

DA6_SetBacks

1. What do you believe are appropriate set-back distances for hydraulic fracturing and related activities from places where people live, including single homes, multiple family dwellings, etc.? Please specify if your response is related to oil or gas extraction.

This is out of the area of my expertise, so I will not speculate. My understanding is that specifying set back distances is essentially a type of risk analysis, and I do not know how to conduct risk analyses.

2. What do you believe are appropriate set-back distances for hydraulic fracturing and related activities from indoor places where people work including offices, hospitals, and schools? Please specify if your response is related to oil or gas extraction.

This is out of the area of my expertise.

For both this question and the previous one as they relate to gas exploration: noise is a major concern. In addition to set backs, sound barriers are available to reduce noise pollution.

3. What do you believe are appropriate set-back distances for hydraulic fracturing and related activities from outdoor places where people work such as farms? Please specify if your response is related to oil or gas extraction.

This is out of the area of my expertise.

4. What do you believe are appropriate set-back distances for hydraulic fracturing and related activities from places where people recreate or play such as parks? Please specify if your response is related to oil or gas extraction.

This is out of the area of my expertise.

5. Should set-back distances differ for settings that include groups of vulnerable individuals, such as schools, day care centers, long- term care facilities, and if so, how? Please specify if your response is related to oil or gas extraction.

This is out of the area of my expertise.

My sense is that the answer should be yes, based on analogy to other pollutant sources. For example, in CA I think the recommended set back for schools is 500 m from the nearest major roadway, whereas residences and businesses can be placed in that 500 m zone.

DA6_SetBacks

Please provide the following information:

Area of professional activity (Check primary activity):

- ☐ X Researcher/scientist
- ☐ Environmental advocate
- ☐ Health care provider
- ☐ Public health professional
- ☐ Other (please specify): ____________________________

Area of expertise (Check primary area):

- ☐ Toxicology
- ☐ X Air quality
- ☐ Water
- ☐ quality
- ☐ Environmental
- ☐ science
- ☐ Environmental health
- ☐ Medicine/health
- ☐ Public health
- ☐ Policy/law
- ☐ Epidemiology
- Social sciences
- Other (please specify): ____________________________

Number of years working in this field: 12________________

Number of years working on issues related to UOGD: 3______________

____

DA7_SetBacks

1. What do you believe are appropriate set-back distances for hydraulic fracturing and related activities from places where people live, including single homes, multiple family dwellings, etc.? Please specify if your response is related to oil or gas extraction.

My responses for this section apply to both oil and gas. Both are often produced and processed in the same locations and involve many of the same processes and equipment. With the exception of a few regions at given periods of time, it can be difficult from a geographic perspective to distinguish between the two. Are there even studies or other documentation that show oil-related activities to have a greater impact than gas-related operations? Certain parts of the production, processing, and transportation/delivery chain may be more emitting or hazardous for oil vs. gas, but it seems hard to say that for the total development chain without more, targeted research.

In general, the closer to the source of pollution (e.g., a well or tank), the greater the potential for exposure to contaminants and the likelihood of impacts to health. Recent studies indicate the potential for exposure at distances of a half-mile or more—far longer distances than requirements in state regulations nationwide. As more science and modeling is done on air contamination, the minimum distance would need to change (and would likely become longer).

[My organization] does not take a position on specific distances, in large part because there is no scientifically definitive distance beyond which health impacts would never occur. However, we believe that current setbacks from residential areas are much too short in all states. Setbacks from water bodies and household water wells are also paltry, from the perspective of risks to ecological systems and drinking water.

Certain distances could be set as a *minimum* setback in regulatory revision efforts, and supported by current science or documentation of contamination, but should not be presumed to then be protective of health. In addition, setbacks from oil and gas development should not be seen as “making it safe”—the industry is inherently polluting and operators must also be required to capture emissions, minimize noise, etc., wherever it operates and whether close or further away from where people live, work, and play.

It is also problematic that state regulations do not distinguish between setbacks from homes and dwellings and other buildings. Clearly setbacks in residential areas should be longer than in industrial areas, where gas operations (and other polluting activities) would be clustered. (This assumes that zoning and planning laws are in place.)

DA7_SetBacks

2. What do you believe are appropriate set-back distances for hydraulic fracturing and related activities from indoor places where people work including offices, hospitals, and schools? Please specify if your response is related to oil or gasextraction.

My response to question #1 applies here as well. People may spend as much or more time at their place of work than at home. Even if people are inside, they go outside and move around their places of work. If you happen to be outdoors when emissions are particularly high or spike (e.g., during flowback, liquids unloading, and compressor station blowdowns), exposure can be intense and harmful. Depending on the type of building in which people work, noise and light can also infiltrate and cause health problems.

3. What do you believe are appropriate set-back distances for hydraulic fracturing and related activities from outdoor places where people work such as farms? Please specify if your response is related to oil or gas extraction.

My response to question #1 applies here as well. However, it is logical to argue for evenlonger setbacks—as well as even more stringent requirements for equipment and procedures—where people and animals are continually outdoors and directly exposed.

4. What do you believe are appropriate setback distances for hydraulic fracturing and related activities from places where people recreate or play such as parks? Please specify if your response is related to oil or gas extraction.

My response to #3 applies here as well.

5. Should set-back distances differ for settings that include groups of vulnerable individuals, such as schools, day care centers, long- term care facilities, and if so, how? Please specify if your response is related to oil or gas extraction.

It is logical to argue for even greater protections for populations that are known to bevulnerable. Earthworks supports the call in Pennsylvania for a “one mile minimum” setback from schools, and to apply this to all settings where vulnerable individuals spend extensive time.

From an advocacy perspective, it makes sense to start with or base calls for longer setbacks on places used or inhabited by people with known vulnerabilities. However, there may be vulnerable individuals living, working, and spending time outdoors even in locations that are not specifically geared toward that population (for example, individuals with compromised immune systems, a history of cancer, or asthma). In addition, exposures to operations that create more health problems can also put more people into the “vulnerable” category over time. This speaks to needing stringent setbacks and operational requirements everywhere.

DA7_SetBacks

Please provide the following information:

Area of professional activity (Check primary activity):

- ☐ Researcher/scientist
- ☐ Environmental advocate
- ☐ Health care provider
- ☐ Public health professional
- ☐ Other (please specify): ____________________

Area of expertise (Check primary area):

- ☐ Toxicology
- ☐ Air quality
- ☐ Water quality
- ☐ Environmental science
- ☐ Environmental health
- ☐ Medicine/health
- ☐ Public health
- ☐ Policy/law
- ☐ Epidemiology
- ☐ Social sciences
- ☐ Other (please specify): ____________________

Number of years working in this field: 20

Number of years working on issues related to UOGD: 5+

Duquesne University IRB
Protocol #2015-08-3
Approved: 9-22-2015
Expiration Date: 9-21-2016

DA8_SetBacks

1. What do you believe are appropriate set-back distances for hydraulic fracturing and related activities from places where people live, including single homes, multiple family dwellings, etc.? Please specify if your response is related to oil or gas extraction.

I do not have an opinion on an appropriate set-back distance because I don't believe there is enough evidence to inform an opinion.

2. What do you believe are appropriate set-back distances for hydraulic fracturing and related activities from indoor places where people work including offices, hospitals, and schools? Please specify if your response is related to oil or gas extraction.

I do not have an opinion on an appropriate set-back distance because I don't believe there is enough evidence to inform an opinion.

3. What do you believe are appropriate set-back distances for hydraulic fracturing and related activities from outdoor places where people work such as farms? Please specify if your response is related to oil or gas extraction.

I do not have an opinion on an appropriate set-back distance because I don't believe there is enough evidence to inform an opinion.

4. What do you believe are appropriate set-back distances for hydraulic fracturing and related activities from places where people recreate or play such as parks? Please specify if your response is related to oil or gas extraction.

I do not have an opinion on an appropriate set-back distance because I don't believe there is enough evidence to inform an opinion.

5. Should set-back distances differ for settings that include groups of vulnerable individuals, such as schools, day care centers, long- term care facilities, and if so, how? Please specify if your response is related to oil or gas extraction.

<u>Vulnerable groups should be considered in establishing health protective set back distances for all upstream energy development.</u>

DA8_SetBacks

Please provide the following information:

Area of professional activity (Check primary activity):

☐ Researcher/scientist
☐ Environmental advocate
☐ Health care provider Public
☐ health professional
☐ Other (please specify): ______________________

Area of expertise (Check primary area):

☐ Toxicology Air
☐ quality Water
☐ quality
☐ Environmental science
☐ Environmental health
☐ Medicine/health Public
☐ health Policy/law
☐ Epidemiology
☐ Social sciences
☐ Other (please specify): ______________________
☐

Number of years working in this field: 28

Number of years working on issues related to UOGD: 5

DA9_SetBacks

1. What do you believe are appropriate set-back distances for hydraulic fracturing and related activities from places where people live, including single homes, multiple family dwellings, etc.? Please specify if your response is related to oil or gas extraction.

Again the distinction between oil and gas is not important. I think there are appropriate, science based set backs that could be developed. I agree with the position that the ones that exist are not science based at all (or only limited science was used, e.g., 150 ft setback based on the height of a drill rig) and are based on political compromises. I am not sure we could do the science in any reasonable time scale, and would encourage people to focus rather on a few key set backs (e.g., schools, hospitals, etc.) but focus on producers ensuring environmental quality and backing that up with continuous monitoring and rigorous enforcement. I would also focus rather on the SIZE of well pads and related infrastructure, as that should be an important consideration in any siting decision and would affect the size of the minimal setback

2. What do you believe are appropriate set-back distances for hydraulic fracturing and related activities from indoor places where people work including offices, hospitals, and schools? Please specify if your response is related to oil or gas extraction.

SEE ABOVE

3. What do you believe are appropriate set-back distances for hydraulic fracturing and related activities from outdoor places where people work such as farms? Please specify if your response is related to oil or gas extraction.

SEE ABOVE

4. What do you believe are appropriate set-back distances for hydraulic fracturing and related activities from places where people recreate or play such as parks? Please specify if your response is related to oil or gas extraction.

SEE ABOVE

5. Should set-back distances differ for settings that include groups of vulnerable individuals, such as schools, day care centers, long- term care facilities, and if so, how? Please specify if your response is related to oil or gas extraction.

DA9_SetBacks

Please provide the following information:

Area of professional activity (Check primary activity):

- ☐ Researcher/scientist
- ☐ Environmental advocate
- ☐ Health care provider
- ☐ Public health professional
- ☐ Other (please specify): ______________________

Area of expertise (Check primary area):

- ☐ Toxicology
- ☐ Air quality
- ☐ Water quality
- ☐ Environmental science
- ☐ Environmental health
- ☐ Medicine/health
- ☐ Public health
- ☐ Policy/law
- ☐ Epidemiology
- ☐ Social sciences
- ☐ Other (please specify): ______________________

Number of years working in this field: 25

Number of years working on issues related to UOGD: 5

[illegible]
Protocol #2015-08-3
Approved: 9-22-2015
Expiration Date: 9-21-2016

DA10_SetBacks

1. What do you believe are appropriate set-back distances for hydraulic fracturing and related activities from places where people live, including single homes, multiple family dwellings, etc.? Please specify if your response is related to oil or gas extraction.

   Setback gas: If these activities have to occur (I propose a total ban), at least two miles

2. What do you believe are appropriate set-back distances for hydraulic fracturing and related activities from indoor places where people work including offices, hospitals, and schools? Please specify if your response is related to oil or gas extraction.

   Setback gas: at least two miles

3. What do you believe are appropriate set-back distances for hydraulic fracturing and related activities from outdoor places where people work such as farms? Please specify if your response is related to oil or gas extraction.

   Setback gas: at least two miles

4. What do you believe are appropriate set-back distances for hydraulic fracturing and related activities from places where people recreate or play such as parks? Please specify if your response is related to oil or gas extraction.

   Setback gas: at least two miles

5. Should set-back distances differ for settings that include groups of vulnerable individuals, such as schools, day care centers, long- term care facilities, and if so, how? Please specify if your response is related to oil or gas extraction.

   Setbacks (gas) should definitely be farther from schools, day care centers where children are located and long-term facilities where people who already have compromised health don't need it further compromised by poor air quality from OG development.

DA10_SetBacks

Please provide the following information:

Area of professional activity (Check primary activity):

Researcher scientist
Environmental advocate
Health care provider
Public Health Professional
Other: ___________________________

Area of expertise (Check primary area):
Toxicology
Air quality
Water quality
Environmental science
Environmental health
Medicine/health
Public health
Policy/law
Epidemiology
Social sciences
Other: ___________________________________

Number of years working in this field: 26

Number of years working on issues related to UOGD: 6

Duquesne University IRB
Protocol #2015-08-3
Approved: 9-22-2015
Expiration Date: 9-21-2016

DA11_SetBacks

1. What do you believe are appropriate set-back distances for hydraulic fracturing and related activities from places where people live, including single homes, multiple family dwellings, etc.? Please specify if your response is related to oil or gas extraction. Minimum of one mile for gas extraction.

2. What do you believe are appropriate set-back distances for hydraulic fracturing and related activities from indoor places where people work including offices, hospitals, and schools? Please specify if your response is related to oil or gas extraction. Minimum of one mile for gas extraction.

3. What do you believe are appropriate set-back distances for hydraulic fracturing and related activities from outdoor places where people work such as farms? Please specify if your response is related to oil or gas extraction. Minimum of one mile for gas extraction.

4. What do you believe are appropriate set-back distances for hydraulic fracturing and related activities from places where people recreate or play such as parks? Please specify if your response is related to oil or gas extraction. Minimum of one mile for gas extraction.

5. Should set-back distances differ for settings that include groups of vulnerable individuals, such as schools, day care centers, long- term care facilities, and if so, how? Please specify if your response is related to oil or gas extraction. Larger setback distances in gas extraction are critical to larger vulnerable groups because one must take into consideration evacuation time and route in case of a catastrophic well or related infrastructure event.

DA11_SetBacks

Please provide the following information:

Area of professional activity (Check primary activity):

- ☐ Researcher/scientist
- ☐ Environmental advocate
- ☐ Health care provider
- ☐ Public health professional
- ☐ Other (please specify): ______________________

Area of expertise (Check primary area):

- ☐ Toxicology
- ☐ Air quality
- ☐ Water quality
- ☐ Environmental
- ☐ science
- ☐ Environmental health
- ☐ Medicine/health
- ☐ Public health
- ☐ Policy/law
- ☐ Epidemiology
- ☐ Social sciences
- Other (please specify): ______________________

Number of years working in this field: 5

Number of years working on issues related to UOGD: 2

DA12_SetBacks

>#1. I do not believe appropriate set-back distances can be determined, as the degree and extent of both surface and air dispersal as well as subsurface dispersal of contaminants and indirect release of toxins (e.g., through pressure bulbs into nearby wells) has not been determined. Research has demonstrated subsurface faulting of up to 1,800 feet from fracturing, and air currents can take volatile compounds large distances, possibly miles, from well pads, storage facilities, and compressor stations (perhaps also from pipelines). Generation of additional underground faulting and measurable earthquake activity at the surface may compound routine subsurface faulting associated with fracturing. Due to our inability, with current information, to predict dispersal pathways accurately, I do not think safe set-back distances can be determined; therefore, most fracking activities (e.g., fracturing itself, storage of flowback and production waters, disposal of waste, transport in compressed pipelines) should not be permitted. If permits are issued, set-backs of at least 1,800 feet would be needed to prevent flow through documented pathways of subsurface contamination. Site-specific research determining wind and other weather patterns might allow determination of appropriate set-backs to prevent air contamination. Studies using "tagged" tracers might further help determine the range of contaminant flows offsite, leading to rational plans for set-backs in specific areas. These considerations apply equally to oil and gas production.
>
>#2. Since health risk increases proportional to length of time of exposure to released toxins, set-backs for places where people spend extended time (home, work, school, hospitals) should be greater than for intermittent exposures. I am not aware of research that demonstrates that indoor exposures are less than outdoor exposures; this might be true for airborne, but not water, exposures.
>
>#3. As indicated in my reply to #4, the amount of exposure outdoors may be greater for airborne contaminants, and workers who are outdoors for prolonged periods, as may happen with farming activities, could have increased exposure. Agricultural exposures may also include an additional route of contact via contaminated dusts and soils, which have accumulated toxins over near-constant airborne exposures or exposure to liquids through leaks or subsurface transport. These considerations apply equally to oil and gas production.
>
>#4. Length of exposure may be considerable in recreational areas such as parks and playgrounds, and contact may occur through multiple routes (airborne, dust, dirt, and possibly water, near streams, ponds, and lakes). While length and extent of exposure may be less than for homes, hospitals, and workplaces, the idea of children and families being exposed during what are expected to be and what are promoted as healthy outdoor activities raises serious ethical questions and suggests that maximal set-backs should apply to recreational areas. These considerations apply equally to oil and gas production.
>
>#5. Populations that are particularly sensitive to the toxins known and suspected to be associated with fracking activities should have special protections; this includes children, neonates, fetuses, embryos, pregnant women, elderly individuals, and those with pre-existing medical or psychological conditions. A set-back distance double the known maximum length of contaminant pathways (i.e, approximately 1 mile) might be considered as a starting point, if exposures cannot be completely eliminated through prohibition of the risky activities. These considerations apply equally to oil and gas production.
>

Duquesne University IRB
Protocol #2015-08-3
Approved: 9-22-2015
Expiration Date: 9-21-2016

DA12_SetBacks

>Area of professional activity (primary): Researcher/scientist (academic research)
>
>Area of expertise (primary): Medicine/health
>
>Number of years working in this field: 33 years
>
>Number of years working on issues related to UOGD: 6 years

Duquesne University IRB
Protocol #2015-08-3
Approved: 9-22-2015
Expiration Date: 9-21-2016

DA13_SetBacks

1. What do you believe are appropriate set-back distances for hydraulic fracturing and related activities from places where people live, including single homes, multiple family dwellings, etc.? Please specify if your response is related to oil or gas extraction. **I am personally of the opinion that there is no safe set-back distance for oil extraction and housing. The appropriate set-back is to leave it "in-the-ground."**

2. What do you believe are appropriate set-back distances for hydraulic fracturing and related activities from indoor places where people work including offices, hospitals, and schools? Please specify if your response is related to oil or gas extraction. **I am of the opinion that offices, hospitals and schools should not be built in the vicinity of oil production. Ambient air quality is compromised in and around the area. VOC's have been known to travel long distances.**

3. What do you believe are appropriate set-back distances for hydraulic fracturing and related activities from outdoor places where people work such as farms? Please specify if your response is related to oil or gas extraction. **We have seen the impact of oil production in close proximity to agriculture. Well casings fail, fluid leaks out, groundwater is contaminated and crops die. Pistachios are the only crop that can appears to be able to withstand the intense salinity leached from produced water. Time will tell.**

4. What do you believe are appropriate set-back distances for hydraulic fracturing and related activities from places where people recreate or play such as parks? Please specify if your response is related to oil or gas extraction. **There are no appropriate set-back distances for recreation areas near oil production. Ambient air quality is affected by VOCs. We have no proof of what constitutes a safe set-back distance. Cumulative effects have yet to be studied.**

5. Should set-back distances differ for settings that include groups of vulnerable individuals, such as schools, day care centers, long- term care facilities, and if so, how? Please specify if your response is related to oil or gas extraction.
Set-back distances should be mandated in local planning and building permits with regard to oil production and vulnerable individuals.

DA13_SetBacks

Please provide the following information:

Area of professional activity (Check primary activity):

- ☐ Researcher/scientist
- ☐ **Environmental advocate**
- ☐ Health care provider
- ☐ Public health professional
- ☐ Other (please specify): ______________________________

Area of expertise (Check primary area):

- ☐ Toxicology
- ☐ Air quality
- ☐ Water quality
- ☐ Environmental science
- ☐ Environmental health
- ☐ Medicine/health
- ☐ Public health
- ☐ Policy/law
- ☐ Epidemiology
- ☐ **Social sciences X**
- Other (please specify): ____

Number of years working in this field: 20

Number of years working on issues related to UOGD: 3

Duquesne University IRB
Protocol #2015-08-3
Approved: 9-22-2015
Expiration Date: 9-21-2016

DA14_SetBacks

1. What do you believe are appropriate set-back distances for hydraulic fracturing and related activities from places where people live, including single homes, multiple family dwellings, etc.? Please specify if your response is related to oil or gas extraction.

    - I'm going to defer to existing regulation that I've come to understand to be fairly well thought-out. The 625' setback from occupied dwellings is probably going to be the most feasible given that many families are going to be leasing their own land (and realizing the financial benefits thereof). I think that the problem may occur when a property owner allows a well to be drilled on their property that then abuts a neighbor's dwelling more closely. If it's on a leasers property who is realizing the financial benefits, I think that the setback is going to be harder to enforce.
    - I'm most familiar with [code]; wells may not be drilled within 250' of an existing water well or developed spring; center of well pads may not be located within 625' of an occupied dwelling structure or a building 2500 sq. ft. or larger used to house or shelter dairy cattle or poultry husbandry; wells may not be drilled within 100' from a perennial stream, natural or artificial lake, pond or reservoir, or a wetland, or within 300' of a naturally reproducing trout stream; no well pad may be located within 1000' of a surface or ground water intake of a public water supply.

2. What do you believe are appropriate set-back distances for hydraulic fracturing and related activities from indoor places where people work including offices, hospitals, and schools? Please specify if your response is related to oil or gas extraction.
    - Again, I'll defer to [code] -- center of well pads may not be located within 625' of an occupied dwelling (I may independently suggest 1250 or ~1/4 mile)

3. What do you believe are appropriate set-back distances for hydraulic fracturing and related activities from outdoor places where people work such as farms? Please specify if your response is related to oil or gas extraction.
    - center of well pads may not be located within 625' of an occupied dwelling structure or a building 2500 sq. ft. or larger used to house or shelter dairy cattle or poultry husbandry

4. What do you believe are appropriate set-back distances for hydraulic fracturing and related activities from places where people recreate or play such as parks? Please specify if your response is related to oil or gas extraction.
    - Given appropriate controls (which can be negotiated in the lease), I believe that a 1000' setback from active outdoor play areas may be appropriate

5. Should set-back distances differ for settings that include groups of vulnerable individuals, such as schools, day care centers, long- term care facilities, and if so, how? Please specify if your response is related to oil or gas extraction.

    - I believe that 2500' or about ½ mile is the distance within which *any* pollution due to natural gas development has been detected. Therefore, I'd recommend this as a setback for vulnerable populations.

DA14_SetBacks

Please provide the following information:

Area of professional activity (Check primary activity):

☐ Researcher/scientist
☐ Environmental
☐ advocate Health
☐ care provider
☐ **Public health**
professional
Other (please specify): ______________________________

Area of expertise (Check primary area):

☐ Toxicolo
☐ gy Air
☐ quality
☐ Water
☐ quality
☐ Environmental
☐ science
☐ Environmental
☐ health
☐ Medicine/health
☐ Public health
Policy/law
Epidemiology
Social sciences
Other (please specify): ______________________________________

Number of years working in this field: 12________________

Number of years working on issues related to UOGD: 4______________

____

DA15_SetBacks

1. **What do you believe are appropriate set-back distances for hydraulic fracturing and related activities from places where people live, including single homes, multiple family dwellings, etc.? Please specify if your response is related to oil or gas extraction.**

2. **What do you believe are appropriate set-back distances for hydraulic fracturing and related activities from indoor places where people work including offices, hospitals, and schools? Please specify if your response is related to oil or gas extraction.**

3. **What do you believe are appropriate set-back distances for hydraulic fracturing and related activities from outdoor places where people work such as farms? Please specify if your response is related to oil or gas extraction.**

4. **What do you believe are appropriate set-back distances for hydraulic fracturing and related activities from places where people recreate or play such as parks? Please specify if your response is related to oil or gas extraction.**

As far away as practicable is my response for questions 1-4. There is some literature available about concentration gradient patterns of particles near highways or other air pollution sources (e.g., see http://www.dustmonitor.com/Articles/Concentration_gradient_patterns_of_aerosol_particles_near.pdf and the references cited in this article). Based on what we understand about air pollution transport, I believe ***at least*** 400 meters (~1/4 mile) setback (and preferably more like a half mile to also address all of the stress related impacts) is needed for all types of hydraulic fracturing and related activities and places where people live, work, and recreate. Even a half mile may not be enough for some types of these operations. My response is related to both oil and gas extraction.

5. **Should set-back distances differ for settings that include groups of vulnerable individuals, such as schools, day care centers, long- term care facilities, and if so, how? Pleasespecify if your response is related to oil or gas extraction.**

As I mentioned in my prior response, as far away as practicable is my response for all of these related questions, and it certainly makes sense to try to increase setback distances for vulnerable populations that are known to be more susceptible to air pollution in particular such as the young, elderly, and those with pre-existing respiratory conditions. (Note, I focused on the air pathway in my responses to this round. There is the potential for water pathway concerns as well, but I feel there is more uncertainty about exposures in that area.) My response is related to both oil and gas extraction.

DA15_SetBacks

Please provide the following information:

Area of professional activity (Check primary activity):

Researcher/scientist Environmental
advocate Health care provider
Public health professional
Other (please specify): ______________________________

Area of expertise (Check primary area):

Toxicology Air quality
Water quality
Environmental science
Environmental health
Medicine/health Public health
Policy/law Epidemiology
Social sciences
Other (please specify): ______________________________

Number of years working in this field: ~4

Number of years working on issues related to UOGD: 23

Duquesne University IRB
Protocol #2015-08-3
Approved: 9-22-2015
Expiration Date: 9-21-2016

DA16_SetBacks

1. What do you believe are appropriate set-back distances for hydraulic fracturing and related activities from places where people live, including single homes, multiple family dwellings, etc.? Please specify if your response is related to oil or gas extraction.

   1000 feet for gas operations based on industry studies of blowouts, explosions and fires from drill rigs, compressor stations and pipelines.

2. What do you believe are appropriate set-back distances for hydraulic fracturing and related activities from indoor places where people work including offices, hospitals, and schools? Please specify if your response is related to oil or gas extraction.

   Indoor sources of stress and reduced productivity include noise and vibration. It is probably not possible to know at this point what an appropriate setback would be to mitigate noise and vibration felt indoors due to highly variable geological conditions, outdoor topography and land use cover, and building construction.

3. What do you believe are appropriate set-back distances for hydraulic fracturing and related activities from outdoor places where people work such as farms? Please specify if your response is related to oil or gas extraction.

   I don’t know.

4. What do you believe are appropriate set-back distances for hydraulic fracturing and related activities from places where people recreate or play such as parks? Please specify if your response is related to oil or gas extraction.

   1000 feet for gas operations based on industry studies of blowouts, explosions and fires from drill rigs, compressor stations and pipelines.

5. Should set-back distances differ for settings that include groups of vulnerable individuals, such as schools, day care centers, long- term care facilities, and if so, how? Please specify if your response is related to oil or gas extraction.

Probably not. It appears that the most vulnerable populations are pregnant women and those with asthma, neither of which would necessarily be concentrated in specific facilities.

DA16_SetBacks

Please provide the following information:

Area of professional activity (Check primary activity):

- ☐ Researcher/scientist
- ☐ Environmental advocate
- ☐ Health care provider Public
- ☐ health professional
- ☐ Other (please specify): ____________________

Area of expertise (Check primary area):

- ☐ Toxicology Air
- ☐ quality Water
- ☐ quality
- ☐ Environmental science
- ☐ Environmental health
- ☐ Medicine/health Public
- ☐ health Policy/law
- ☐ Epidemiology
- ☐ Social sciences
- ☐ Other (please specify): ____________________
- ☐

Number of years working in this field: 15

Number of years working on issues related to UOGD: 3

DA17_SetBacks

1. What do you believe are appropriate set-back distances for hydraulic fracturing and related activities from places where people live, including single homes, multiple family dwellings, etc.? Please specify if your response is related to oil or gas extraction.

2 KM from natural gas wells

2. What do you believe are appropriate set-back distances for hydraulic fracturing and related activities from indoor places where people work including offices, hospitals, and schools? Please specify if your response is related to oil or gas extraction.

2 KM

3. What do you believe are appropriate set-back distances for hydraulic fracturing and related activities from outdoor places where people work such as farms? Please specify if your response is related to oil or gas extraction.

2 KM

4. What do you believe are appropriate set-back distances for hydraulic fracturing and related activities from places where people recreate or play such as parks? Please specify if your response is related to oil or gas extraction.

2 KM

5. Should set-back distances differ for settings that include groups of vulnerable individuals, such as schools, day care centers, long- term care facilities, and if so, how? Please specify if your response is related to oil or gas extraction.

2 KM

DA17_SetBacks

Please provide the following information:

Area of professional activity (Check primary activity):

- ☐ XResearcher/scientist
- ☐ Environmental
- ☐ advocate Health care
- ☐ provider Public health
- ☐ professional

Other (please specify): ______________________________

Area of expertise (Check primary area):

- ☐ Toxicology
- ☐ Air quality
- ☐ Water
- ☐ quality
- ☐ Environmental
- ☐ science
- ☐ X Environmental
- ☐ health
- ☐ Medicine/health
- ☐ XPublic health
- ☐ Policy/law

Epidemiology
Social sciences
Other (please specify): ______________________________

Number of years working in this field: 15_______________

Number of years working on issues related to UOGD: 4_____________

Duquesne University IRB
Protocol #2015-08-3
Approved: 9-22-2015
Expiration Date: 9-21-2016

DA18_SetBacks

1. I do not believe this is calculable.

2. I do not believe this is calculable.

3. I do not believe this is calculable.

4. I do not believe this is calculable.

5. Vulnerable populations are distributed throughout the environment. This is therefore an inadequate calculation to consider.

Please provide the following information:

Area of professional activity (Check primary activity):

Researcher scientist
Environmental advocate
Health care provider
Public Health Professional
Other: [personal identifier]

Area of expertise (Check primary area):

Toxicology
Air quality
Water quality
Environmental science
Environmental health
Medicine/health
Public health
Policy/law
Epidemiology
Social sciences
Other: risk analysis and management

Number of years working in this field: Environmental health and public health—17 years; environmental science 23

Number of years working on issues related to UOGD: 6
